# Editorial: Evolution of Organismal Form: From Regulatory Interactions to Developmental Processes and Biological Patterns

**DOI:** 10.3389/fgene.2016.00148

**Published:** 2016-08-17

**Authors:** Sylvain Marcellini, Hector Escriva

**Affiliations:** ^1^Laboratory of Development and Evolution, Department of Cell Biology, Faculty of Biological Sciences, Universidad de ConcepciónConcepción, Chile; ^2^Observatoire Océanologique de Banyuls sur Mer, Centre National De La Recherche Scientifique, UPMC Université Paris 06, UMR 7232, BIOMBanyuls sur Mer, France

**Keywords:** Evo-Devo, regulatory interactions, developmental processes, biologiccal patterns, genomes

Living organisms display an astonishing morphological and behavioral diversity shaped by extrinsic environmental conditions and by intrinsic changes in developmental processes. In turn, such developmental trajectories are contingent on a myriad of regulatory interactions occurring at all possible steps of gene expression and cellular function. We are pleased to present a Frontiers Research Topic composed of 10 original research articles and reviews whose focus, ideas, and hypotheses reflect the current diversity and future directions of the field of Evo-Devo.

The evolution of gene families, gene expression patterns, and alternative splicing are addressed by examining germline determinants in cephalochordates and their implications for our understanding of multipotency and regeneration (Dailey et al.), by an extensive analysis of Fox members expressed during amphioxus early embryogenesis (Aldea et al.), and by deciphering the origin and expansion of alternative splicing of Pax genes in chordates (Fabian et al.). The evolution of cell types and tissue morphogenesis are addressed by illustrating, using the insect extraembryonic epithelia, how tissue architecture and physical context are crucial to understand gene function and evolution (Horn et al.), by proposing a model to explain how the transition between immature and mature cartilage might have facilitated the emergence of the osteoblastic regulatory network (Gomez-Picos and Eames), and by examining major fibrillary collagen genes expressed in the catshark and the clawed frog skeletons, thereby providing new insights on the origin of cartilage calcification (Enault et al.). Finally, some authors discuss important concepts in the field, such as the interpretations of heterologous assays and their possible pitfalls (Kramer), the convergent evolution of a two-steps morphogenetic mechanism controlling organ shape in plants and animals (Mentink and Tsiantis), the evolution of short peptide motives driving the generation of specific protein complexes involved in key bilaterian innovations (Merabet and Galliot), and the intricate relationships linking the genotypic and phenotypic dimensions (Orgogozo et al.).

So, what is the broad contribution to Evo-Devo of the 10 aforementioned manuscripts, and how do they relate to the future research directions that this discipline must prioritize in order to remain both successful and attractive to the community? Hints to our first answer are included within the topic title itself, inspired from a François Jacob influential review emphasizing the complex and crucial relationships between different levels of biological organization (Jacob, 1977). Undoubtedly, understanding how specific mutations and environmental factors affect molecular networks, cells, organs, species, and ecosystems, represents one of the most stimulating Evo-Devo conceptual frameworks. While it is a long way to connect “regulatory interactions to developmental processes and biological patterns,” the initial and obligatory step is to report expression patterns of key molecular actors (Aldea et al.; Fabian et al.; Dailey et al.). One can then move to higher hierarchical levels, for instance by understanding the mechanisms facilitating the emergence of regulatory interactions involved in body plan patterning (Merabet and Galliot), or by integrating gene activity, cellular behavior and mechanical forces to reach a comprehensive view of embryonic evolution (Horn et al.). Our second answer relates to our ability to cope with a modern era continuously flooded by ever-improving imaging, genome editing and sequencing technologies. As a consequence, data analysis, and not data generation, should be our present priority (Moore, 2012). As recently argued, facing the next grand challenge in evolutionary biology will require a strong synergy between three major branches of the field: Experimental data, genomics, and modeling (Cushman, 2014). According to this strategy, evolutionary models must be validated by performing carefully designed and controlled experiments, which, in the case of heterotopic functional assays, should follow the guidelines proposed by Kramer. This multidisciplinary approach will be particularly well-suited to extract universal principles underlying the development of multicellular organisms (Mentink and Tsiantis), to decipher the evolution of the bone and cartilage gene regulatory networks (Enault et al.; Gomez-Picos and Eames), or to understand, for any species of interest, how the interaction between genotype and environment generates complex phenotypic spaces (Cushman 2014; Orgogozo et al.). These are exciting times for the Evo-Devo community, we hope that you will enjoy this collection of articles and look forward in the near future to reading any follow-up work that it will have inspired.

## Author contributions

SM and HE wrote, edited, and revised the manuscript.

### Conflict of interest statement

The authors declare that the research was conducted in the absence of any commercial or financial relationships that could be construed as a potential conflict of interest.
